# Procedural pain in pediatric care: educational, ethical, and public health imperatives

**DOI:** 10.3389/fped.2026.1818666

**Published:** 2026-05-07

**Authors:** Christoffer Krug, Michael Knipper, Philipp Stieger

**Affiliations:** 1 Center for Outpatient Pediatric and Adolescent Medicine, Academic Educational Practice of the Faculty of Medicine, Justus Liebig University Giessen, Giessen, Germany; 2 Institute for the History, Theory and Ethics of Medicine, Justus Liebig University Giessen, Giessen, Germany; 3 University Clinic for Cardiology and Angiology, Otto-von-Guericke University Magdeburg, Magdeburg, Germany

**Keywords:** ethical standards of care, medical education, needle fear, pain prevention, pediatric procedural pain, public health impact

## Introduction

A recent Editorial in Lancet Child Adolescent Health focuses on the diverse forms of daily violence experienced by children worldwide and outlines their long-term consequences (1). The authors emphasize that such harm is preventable but requires sustained implementation and prioritization of effective interventions across systems of care and that these efforts remain insufficient in practice. A similar gap exists in pediatric procedural pain, where evidence-based strategies are available but inconsistently applied. This routine exposure of children to avoidable pain reflects systemic failures and can even be understood as a form of structural violence within the health care system (2). Despite a substantial body of evidence and existing guidelines, pediatric procedural pain remains inconsistently addressed in clinical practice. This discrepancy raises the question of why effective interventions are not systematically implemented and what structural factors sustain this gap.

Needle-related procedural pain represents one of the most frequent recurring sources of distress in pediatric care. National and international guidelines explicitly define inadequately treated procedural pain as a care deficit, especially in patients who have limited ability to articulate pain (3–6). In this context, children represent a particularly vulnerable population, as they are dependent on the anticipatory perception and protection of healthcare professionals. Although procedural pain is not deliberately inflicted, it is often tolerated and thus effectively normalized (7). This discrepancy between normative requirements and clinical reality is particularly evident in medical education and training, where procedural pain management has so far only been incorporated into the curriculum to a limited extent (8, 9).

Routine procedures such as vaccinations, venipunctures, or the insertion of intravenous catheters repeatedly expose children to pain, fear, and anticipatory tension (10). These experiences often begin early in life and are repeated throughout childhood. A growing body of evidence shows immediate and long-term consequences, including increased sensitivity to pain, fear of medical procedures, and phobias with a prevalence of approximately 20%–50% (11–15). In addition, reduced trust in medical professionals and reduced use of preventive health measures are described (16).

This article examines pediatric procedural pain from a medical education, ethical, and public health perspective and takes up the demand made in current specialist articles that every child has a right to adequate pain relief and that effective, evidence-based interventions are available (17). In this article, we aim to address this gap by synthesizing existing evidence and interpreting it through a conceptual framework that links structural violence, the hidden curriculum in medical education, and a rights-based approach to child health.

When effective pain prevention strategies are available but systematically not implemented, the resulting harm is not accidental but structurally produced. In this sense, untreated procedural pain in children can be understood as a form of structural violence—embedded in routine practices, institutional priorities, and educational omissions rather than individual intent. This opinion article reframes pediatric procedural pain as a systemic failure and provides the basis for examining the problem from educational, ethical, and public health perspectives.

## Evidence base

Over the past 20 years, extensive evidence has been gathered showing that simple, inexpensive, and easily implemented interventions can significantly reduce pain and distress during pediatric needle procedures (3, 18–20). These include pharmacological strategies such as topical local anesthetics and multimodal non-pharmacological measures such as physical aids, oral sucrose or breastfeeding in infants, age-appropriate distraction and communication techniques, and comfort-promoting positioning in the presence of caregivers (18–20). Nevertheless, this evidence has not yet been implemented across the board in practice. The lack of systematic implementation is therefore less a problem of knowledge and more a problem of structure, training and responsibility. This observation forms the basis for the following analysis, which examines how structural, educational, and normative factors interact to sustain this implementation gap.

## Reasons for the lack of implementation

Despite clear evidence and good feasibility, the application of pain prevention measures remains inconsistent. Particularly in busy outpatient settings and hospital wards, time pressure, staff shortages, communication problems, and established routines lead to pain management being neglected (21–23). This implementation gap cannot be explained solely by a lack of resources, but also reflects implicit norms and patterns of thinking about procedural pain that persist in everyday clinical practice (24, 25). These require critical reflection among healthcare professionals. Importantly, these factors do not operate in isolation but reinforce each other within institutional routines and professional socialization processes, thereby stabilizing the persistence of avoidable pain.

Procedural pain is often normalized and trivialized in communication, for example through minimization or narratives of the inevitability of pain (26). As a result, pain treatment may be perceived as an optional addition that disrupts procedures, rather than as an integral standard of care (27). Professional norms and implicit assumptions about children's pain, including enduring “pain myths”, may influence how pain relief is prioritized in routine care (28). Guidelines also show that trivializing or dishonestly reassuring language is of no benefit and can be associated with increased distress and loss of trust (29). These patterns contribute to the persistence of avoidable pain in routine practice despite available interventions.

## Educational perspective

Persistent undertreatment of pediatric procedural pain reflects a structural deficit that begins in undergraduate medical education and extends through postgraduate and specialist training. While technical skills for invasive procedures are taught, structured, longitudinal, and interprofessional instruction in pain prevention, child-centered communication, and evidence-based non-pharmacological strategies remain largely absent and rarely formally assessed, reflecting a hidden curriculum (24). The concept of the hidden curriculum is particularly useful here, as it describes how implicit norms and practices shape professional behavior beyond formal teaching, thereby contributing to the normalization of avoidable pain early in professional socialization and ongoing across training stages. The lack of clearly defined mandatory learning objectives signals that pain prevention is not recognized as a core professional obligation. Competencies that are neither systematically taught nor evaluated are implicitly framed as optional, reproducing a hidden curriculum in which children's distress is tolerated rather than prevented (24). From this perspective, the omission of structured pain education is not merely a curricular gap but a mechanism through which systemic shortcomings are reproduced across generations of healthcare professionals. Recognizing children's rights to protection from unnecessary suffering requires integrating pain prevention into medical curricula and postgraduate training, supported by binding learning objectives, interprofessional teaching, and institutional accountability. Without enforceable standards, educational initiatives risk remaining symbolic rather than transformative.

## Accountability and governance

The persistent gap between evidence and practice in pediatric procedural pain management cannot be explained by individual knowledge deficits alone. As long as pain prevention remains optional rather than mandated, responsibility is effectively displaced from institutions to individual clinicians. This lack of institutional accountability allows avoidable pain to persist as a normalized by-product of routine care. This shift from individual responsibility to institutional accountability represents a key structural intervention to address the implementation gap identified throughout this article.

Embedding pain prevention into binding clinical standards, institutional policies, and quality assurance mechanisms, including documentation requirements and regular audits, is therefore essential to shift responsibility from individual discretion to systemic obligation.

## Ethical perspective

Pediatric procedural pain raises fundamental ethical questions. Depending on their age, children may not be able to give reliable informed consent and often lack the ability to adequately communicate pain. Worldwide infants and toddlers represent a “silent” population without a political or institutional lobby. Their suffering often remains unrecognized precisely because it is transient, episodic, and embedded in routine care processes. Furthermore, this structural vulnerability reflects broader social and institutional determinants of health, which affect access to adequate care and protection from preventable harm and highlights the importance of a rights-based and structural approach to health inequities (30). The concept of structural violence provides a useful ethical lens in this context, as it captures how harm can arise not from individual intent but from systematically organized practices and omissions within healthcare systems.

Repeated exposure to avoidable pain is in fundamental conflict with core ethical principles such as non-maleficence, care, and respect for human dignity. The failure to take effective, available pain relief measures despite sufficient evidence constitutes an inadequate standard of care (29). These ethical considerations are closely linked to human rights frameworks: international conventions on the rights of the child emphasize the right to the highest possible level of health and protection from unnecessary suffering (31, 32). A rights-based framework further strengthens this argument by linking clinical practice to internationally recognized obligations to protect children from preventable harm. The systematic toleration of avoidable pain can therefore be interpreted as a systemic failure to uphold these rights.

Similarly, certain adult patient groups are also vulnerable to inadequate pain management, especially if they are unable to reliably articulate pain verbally, such as older people with dementia or people with severe neurological impairments (33). Studies show that pain in cognitively impaired adults is often less well recognized and treated, highlighting the structural dimension of the problem (34).

## Public health perspective

From a public health perspective, pediatric procedural pain is of considerable relevance. Negative early medical experiences are consistently associated with the development of needle phobia, avoidance behavior towards the healthcare system, and later vaccine hesitancy (11, 35, 36).

Needle phobia thus represents a relevant barrier to preventive health services and influences population-based health goals (37). These findings underscore that pediatric procedural pain is not only a clinical or ethical issue but also a population-level determinant of health behavior, linking early experiences of care to long-term public health outcomes.

Pediatric procedural pain prevention can therefore be understood as an early investment in population health behavior. Failing to adequately treat avoidable pain compromises not only immediate well-being but also long-term trust, adherence, and engagement with other preventive health services (17). In addition, procedural pain and pronounced fear reactions in children are also perceived as emotional stress by medical staff and are associated with increased stress and burnout risk (36, 38).

## Conclusion

Given the persistent structural deficit in pediatric pain education, the continued marginalization of procedural pain prevention across undergraduate and postgraduate training is no longer justifiable. Training programs must explicitly integrate evidence-based pharmacological and non-pharmacological strategies, age-appropriate communication, child-centered procedure planning, and systematic caregiver involvement. These competencies should be longitudinally embedded in curricula, taught across professional groups, and institutionally enforced, with mandatory learning objectives and structured assessment formats (e.g., OSCE, DOPS, Mini CEX). Needle- and procedure-related pain in children is not an unavoidable side effect of medical care but a preventable, evidence-based problem with profound clinical, ethical, and public health implications. Taken together, the arguments presented in this article suggest that pediatric procedural pain should be understood as a paradigmatic example of a preventable yet normalized form of harm, sustained by interacting structural, educational, and normative factors. Structural inattention to pain prevention early in professional education perpetuates a culture in which children's distress is tolerated rather than systematically prevented, and similar normalization processes affect other patients with limited communicative capacity (e.g., sedated, cognitively impaired, or critically ill adults). Every painful procedure is a formative moment that either strengthens or undermines trust in healthcare. By explicitly integrating the concepts of structural violence, hidden curriculum, and a rights-based approach, this article contributes a conceptual framework that helps explain why existing evidence has not translated into routine practice. Embedding pediatric pain prevention as a mandated standard of care across education and practice is essential to ensure clinically effective, ethically responsible, and rights-based care. Strengthening this perspective requires not only awareness but also enforceable structural changes that align clinical practice with existing evidence and ethical obligations. Without binding institutional standards, longitudinal training, and accountability, avoidable pain will remain normalized, compromising children's well-being and their fundamental right to health.
